# First-line managers’ experience of guideline implementation in orthopaedic nursing and rehabilitation: a qualitative study

**DOI:** 10.1186/s12913-024-11353-w

**Published:** 2024-07-31

**Authors:** Erika FJORDKVIST, Maria HÄLLEBERG NYMAN, Madeleine WINBERG, Eva JOELSSON-ALM, Ann Catrine ELDH

**Affiliations:** 1 Faculty of Medicine and Health, School of Health Sciences, Örebro University, 701 82 Örebro, Sweden; 2 Department of Orthopaedics, Faculty of Medicine and Health, Örebro University, 701 82 Örebro, Sweden; 3 Faculty of Medicine and Health, University Health Care Research Center, Örebro University, 701 82 Örebro, Sweden; 4 Faculty of Medicine and Health Sciences, Department of Health, Medicine and Caring Sciences, Linköping University, 581 83 Linköping, Sweden; 5 Department of Clinical Science and Education, 118 83Stockholm, Södersjukhuset, Karolinska Institutet Sweden; 6 Department of Public Health and Caring Sciences, Uppsala University, Box 564, 751 22 Uppsala, Sweden

**Keywords:** Clinical practice guideline, Evidence-based health care, Evidence-based nursing, Evidence-based practice, First-line manager, Implementation, Implementation leadership

## Abstract

**Background:**

First-line managers have a unique role and potential in encouraging the use of evidence-based clinical practice guidelines (CPGs) and thus serve the provision of safe patient care. In acute and planned hospital care, effective yet safeguarded nursing procedures are a necessity. Little is currently known about how first-line managers engage in supporting the adoption of evidence-based nursing care and about what barriers and enablers there are for implementation of CPGs in the orthopaedic care context.

**Purpose:**

To investigate first-line managers’ experience of clinical practice guideline implementation in orthopaedic care.

**Methods:**

This qualitative interview study included 30 first-line nursing and rehabilitation managers in 17 orthopaedic units in Sweden. A deductive content analysis, with the Ottawa Model of Implementation Leadership as a guide, was employed.

**Results:**

To the first-line managers, any guideline implementation required them to balance contexts, including their outer context (signified by the upper-level management and decision-makers) and their inner context, including staff and patients in their unit(s). Acting in response to these contexts, the managers described navigating the organization and its terms and conditions; using relations-, change-, and task-oriented leadership, such as involving the staff; motivating the change by emphasizing the patient benefits; and procuring resources, such as time and training. Even though they knew from past experience what worked when implementing CPGs, the first-line managers often encountered barriers within the contexts that hampered successful implementation.

**Conclusions:**

Although first-line managers know how to effectively implement CPGs, an organization’s terms and conditions can limit their opportunities to fully do so. Organizational awareness of what supports and hinders first-line managers to offer implementation leadership can enhance opportunities to alter behaviours and conditions for the benefit of CPG implementation.

**Trial registration:**

The study was registered as NCT04700969 with the U.S. National Institutes of Health Clinical Trials Registry on 8 January 2021.

## Background

Healthcare leaders are identified as key in facilitating evidence-based practice (EBP) [1, 2]. A vital source of safe, evidence-based nursing procedures is evidence-based clinical practice guidelines (CPGs) [3], although the implementation of such CPGs varies greatly. It is estimated that as much as 30–40% of care delivered is not sufficiently evidence-based [4, 5]. With no exact details on the know-do gap in nursing, knowledge implementation is known to vary [6]. The growing field of implementation science addresses barriers and enablers for EBP, shedding light on strategies to facilitate and sustain, for example, CPG adoption and adherence [6, 7], although there is a need for further investigation of leadership components in knowledge implementation [2, 8].

First-line managers (i.e., those who directly supervise staff providing care and who are, in turn, supervised by a superior manager) [9] are essential to both identifying and addressing the barriers and enablers for safer and better care [10, 11]. In nursing care, they can enable nurses to use their competencies to enhance EBP [12] and to de-implement outdated healthcare procedures [13]. Although first-line managers should enact implementation leadership, what this encompasses is not fully known [14], particularly in acute care settings, with their high turnover of patients.

Orthopaedic surgical care is associated with both fundamental and advanced nursing and the rehabilitation needs of patients. In hospitals around the world, approximately 1.7 billion people are cared for every year due to musculoskeletal conditions [15]. Several orthopaedic surgical treatments have more recently been associated with shorter length of stay [16, 17], calling for effective yet safeguarded care, including nursing care based on and reflecting EBP. Consequently, the orthopaedic context, that is, the milieu in which patients with musculoskeletal problems are cared for, is associated with numerous challenges and opportunities.

With respect to knowledge implementation, context is defined as “the environment or setting in which the proposed change is to be implemented” ([18] p.150). Context refers to a variety of dimensions, such as organizational support, organizational culture and climate, leadership, and financial resources, as well as social relations and support, physical environment, and patient preferences. Context is divided into different system levels: macro-, meso-, and micro-level. These are often grouped into descriptions as an outer or an inner context, even though the line between the two is not always clear. Context includes dimensions representing essential conditions for implementation as well as functions that drive the implementation towards its goal [19]. Little is currently known regarding what barriers and enablers in the orthopaedic context hinder and facilitate optimal nursing and rehabilitation. However, a previous study indicated a lack of leadership support, including management actions and attitudes, when it comes to CPG implementation [20]. Thus, there is a need to further address what options first-line managers have to support knowledge implementation, and how they proceed.

## Method

### Aim

To investigate first-line managers’ experience of clinical practice guideline implementation in orthopaedic care.

### Design

A descriptive, qualitative interview study [21], reported with respect to the Consolidated Criteria for Reporting Qualitative Research (COREQ) [22].

### Setting

This study was performed within the Onset PrevenTIon of Orthopaedic Nursing and rehabilitation project, OPTION [23], enacted across 17 Swedish orthopaedic units. At the onset of the trial, we performed individual interviews with all of the units’ first-line managers (February–August 2021), including university, regional, and local hospitals.

### Recruitment and participants

At each site, all nursing and rehabilitation managers were identified and invited to partake in an OPTION interview. The interviews were conducted by EF or MW; neither of the interviewers were involved in the subsequent OPTION intervention, and both were blind to the randomization outcome at the point of the interviews. Altogether, 35 eligible managers were contacted via email or telephone; four rehabilitation managers declined due to a heavy workload, resulting in 31 individuals consenting to participate in the study. As one interview was lost due to technical problems with the recording, the study includes interviews with 30 managers (at least one and often two managers from each site). Demographics are presented in Table 1. The nursing managers were registered nurses (except one, who was a physiotherapist), while the rehabilitation managers were physiotherapists or occupational therapists. Participants’ experience as managers varied: some had been managers in several workplaces prior to their current position and others were in their first management role. Most of the first-line managers had full-time management positions, but two had part-time clinical practice scheduled. Yet all the first-line managers described helping out with clinical practice when they had the opportunity or if there was a staff shortage.

**Table 1:** Demographic characteristics of the first line managers

**Variables**	(*N* = 30)
**Age, median ** ***(min–max)***	49.5 (38–66)
**Sex, n (%)**	
Female	28 (93.3)
Male	2 (6.7)
**Type of manager, n (%)**	
Nursing manager	14 (46.7)
Rehabilitation manager	13 (43.3)
Both nursing and rehab manager	3 (10.0)
**Managing experience, years, median (min–max)**	5 (0.5–24)
**Relevant educations, n (%)**	
Specialist training	7 (23.3)
Master education	2 (6.7)
Doctoral degree	1 (3.3)
Leadership education (internal and external)	12 (40.0)
Quality assurance course	1 (3.3)
Health care administration	1 (3.3)

### Data collection

Data was collected through semi-structured interviews based on a previously validated interview guide [24]. The guide indicated a narrative approach with the following areas of interest: experiences of nursing/rehabilitation care; management experience(s); development and in-house training opportunities; the organization and its structures and processes; change management and practice; improvement initiatives and performance; and outcomes and feedback. Probing questions were used sparingly to encourage further discussion. Prior to the interviews, the interview guide was discussed thoroughly between all authors to ensure a shared understanding and equivalent approach in all interviews. EF or MW held the telephone interviews, at a time chosen by each first-line manager.

The interviews lasted between 25 and 68 min (median 47 min), and all exhausted every aspect of the interview guide; they were digitally recorded in full and transcribed verbatim by an authorized secretarial service, resulting in 348 single-spaced pages of transcribed text.

### Data analysis

Data consisted of the transcribed interviews which were analysed with qualitative content analysis [25], inspired by phenomenological hermeneutics [26] for a broadened understanding of managers’ lived experience vis-à-vis implementation of CPGs as a phenomenon.

First, all interviews were read in their entirety, and all authors individually compiled a short text representing their naïve understanding. The fundamentals were discussed until agreement was reached on the common content of the data set, providing a backdrop for the further analysis [26].

Second, the structured analysis applied a deductive approach [25], using categories from the Ottawa Model of Implementation Leadership (O-MILe) [27] as a matrix: *core knowledge and skills; change-oriented leadership; relations-oriented leadership;* and *task-oriented leadership*. In this phase, all interviews were reread several times; meaning units were identified and organized in accord with the matrix, using NVivo software (version 1.3) [28].

The deductive analysis progressed by means of a critical inspection of the leadership components and structures described in O-MILe. In this phase, the naïve understanding was used to anchor the analysis, investigating nuances of the process description in O-MILe and beyond. Data that did not match the O-MILe categories but corresponded to the study aim was analysed inductively, forming additional perspectives. These were either additional aspects of managing change or characteristics of the context influencing the implementation process.

Altogether the initial findings were subjected to an abductive analysis, guided by queries such as: *what is shared (i.e., what is the managers’ experience of guideline implementation), by whom, in what context, and why?* [25].

To conclude, a comprehensive understanding, demonstrating the final outcomes of the analysis, was formed [26].

### Rigour

While the analysis was primarily performed by the first author (EF) guided by the last author (ACE), all steps were repeatedly discussed between all authors and critically surveyed to compare maturing understandings of the data set at each of the phases. In alignment with criteria for qualitative research [22], trustworthiness (that is: credibility; dependability; conformability; transferability, and; authenticity) was considered in the preparation for and completion of the analysis, as well as in the organization and reporting of the results [29]. A selection of quotations illustrating the route to categories in the analysis is provided in Table 2 [30].

**Table 2:** Illustration of the analysis, from quotations to category (in alphabetical order of the latter)

Quotation, equaling meaning unit (with numerical code for which interview)	Illustrating which category
We need to learn how to raise our voice and not just sit there with the feeling of being unheard. If they [upper level management] don’t listen, we need to find other ways. (Manager 12)	Act and react to support change
I lack a clear structure [for CPG implementation]. Now, it’s like a jungle and often, it is up to us managers to operate [change]. (Manager 9)	
Large-scale changes often face challenges due to the need for collaboration across departments. Misalignment of goals or misunderstandings about objectives can lead to discouragement and abandonment of the change. (Manager 8)	Balancing contexts
The decision-making process can be chaotic at times, with directives coming from various directions. Political decisions are made that require implementation, but we have multiple stakeholders like physicians, managers, and politicians. (Manager 30)	
It is necessary to create an understanding of the purpose of the change and to see the benefits for both the patient and oneself. (Manager 10)	Change-oriented leadership behaviours
It’s really important to create an understanding of why the change is made. (Manager 4)	
I firmly believe in communication and involving employees from the outset, ensuring they feel empowered to contribute even to the smallest details. In other words, we should design this together, fostering a sense of participation among them. (Manager 26)	Creating a plan, acting from learned experiences of what works
In general, I think there's a certain amount of politics involved when it comes to change. When you can involve the affected staff at an early stage, and devote a lot of time to talking about it, or giving them a chance to have their say, implementation tends to be smoother. (Manager 3)	
It’s hard when you don’t get the time to understand why the change is necessary, when directives just came from above [upper management]. (Manager 14)	Directives to implement guidelines
When it comes to top-down guideline [implementation], it's not much we as a unit can do, but rather something we have to adapt to. (Manager 24)	
Convincing employees to embrace new ideas or changes is an art form. (Manager 11)	Marketing guidelines to inner context
I strive to be responsive to feedback, appraising whether my ideas are useful for the staff. Ultimately, we collectively decide whether to adopt or discard such ideas. (Manager 19)	
The decision-making process is often sluggish and bureaucratic, resulting in a frustrating lack of progress and approvals. (Manager 27)	Navigating the organization and its terms and conditions
It almost becomes a political agenda, huh, but I would say nursing is not visible enough. And that's something we need to work on a lot. We started making this comical video on the ward […], showing patient recovery with and without nursing care after a hip replacement. Then the hospital’s legal entity banned any filming in the ward. (Manager 20)	
So, finances, decisions, national guidelines, and interest all play a significant role. However, staffing is a major factor, as are resources. Without adequate resources, I don't think we have the energy to tackle the issues we know about and have ideas for addressing. But if we don't have the time, then it's difficult as well. (Manager 23)	Outer and inner context
So, the patient turnover here is incredibly fast, and once patients have undergone surgery, we send them off to rehab, geriatrics, or their own homes. (Manager 1)	
I’m also creating like an explanatory frame; I understand the importance of clarifying ‘how this will work and why’. In this process, information is crucial. (Manager 22)	Relations-oriented leadership behaviours
I've really needed to recognize the employees' skills in different areas and provide opportunities for them to do a good job. (Manager 7)	
I set aside dedicated time each week for employees to stay up-to-date [with research]. (Manager 16)	Task-oriented leadership behaviours
You have to provide training, and in several cases, you need to repeat that training. (Manager 28)	
However, if it's a process that's being worked on at the [upper] management level, then once it's finalized, the operational managers receive it and are supposed to pass it on to us team leaders. But it is also advertised in this management letter that the hospital director publishes, so we get it that way too. And then it's up to me to disseminate it to my staff and ensure that the guideline is followed. (Manager 21)	Translating guidelines to fit the inner context
If the routines come to involve other units, we have to cooperate to adjust such routines together. (Manager 2)	
We must rely on information trickling down from [upper] management and that they keep us informed about developments. (Manager 13)	Trusting directed guidelines to be correct
However, overall, we are quite—I mean, we are very much guided by pro memoria and per protocols, and we are very structured in that way. I actually think that's a good thing. (Manager 25)	
As a manager, I have to be assertive about the change, and at the same time give room for the staff to be involved. (Manager 5)	Using a variety of leadership behaviours
Before I present another change, I evaluate the state of my staff – are they ready for a change? By doing this, I get them more involved and get better results. (Manager 17)	
When implementing major changes, we conduct a thorough mapping of the patient pathway and identify their needs along the way, in collaboration with other significant units [of the hospital]. (Manager 6)	Vison of a better patient care
Patient involvement is a crucial driver for creating lasting and genuine change. (Manager 10)	

## Results

The results are presented in the following order: the outcomes of the structured analysis followed by the comprehensive understanding. For each section, the categories from O-MILe are  bolded, and supplementary categories are italicized. 

### The structured analysis’ outcomes

*Directives to implement guidelines* most often came from the *outer context* (described as the upper-level management, authorities, politicians, and sometimes individual physicians), and they were issued with no room for questioning as to when and how implementation should take place. The first-line managers described their mission as to implement the directed guidelines in the* inner context*, which was their unit, with the staff they supervise. The managers sometimes worried that a proposed implementation might not fit well in the inner context when they sensed that no proper risk assessment had been done to identify potential consequences for their units. The first-line managers did not always know exactly who had made the decision about a guideline prior to its implementation; they simply described the directives as coming “from above”. With no or limited opportunities to discuss the guidelines to be implemented, they still initiated the implementation by means of communication. The first-line managers described prioritizing *marketing guidelines to the inner context* (i.e., to their staff and unit(s)) while also figuring out how they could be *translated to fit the inner context*. By protecting their inner context, the first-line managers shielded the staff from petitions coming from the outer context, which could otherwise create stress and anxiety in their units. As the first-line managers indicated, the healthcare organizations were not functioning optimally at all times, making it hard to implement changes. However, this was often due to a lack of communication between units or other parts of health care. The first-line managers described adjusting actions in the process, considering whom they interacted with. They described using acceptance and adjustment and emphasizing their trust in decisions from top management in order to move forward with the implementation of guidelines in the inner context.

Adjusting actions in an implementation process included *using a variety of leadership behaviours*. The first-line managers emphasized the necessity of *creating a plan, acting from learned experiences of what works*, and they described the importance of informing and involving the staff early in the process, corresponding to **relations-oriented leadership**. The first-line managers described how they emphasized the patient perspective when communicating the reasons for adopting a new guideline, suggesting that this was a motivator for any change. Even with a *vision of better patient care*, the first-line managers indicated that they did not make enough effort to involve patients in the change process. They raised this as a limitation as, in their view, patient involvement in health care should be a higher priority. Being well rehearsed when it came to knowing the staff and the clinical routines, the first-line managers described relying on their insights into the culture and attitudes when addressing a change such as guideline implementation. To some extent, the first-line managers relied on staff members who were positive towards change in general to be facilitators when new routines or specific changes were introduced. However, there was a risk that the new routines may be dropped should those positive co-workers leave. If the professionals displayed a negative attitude towards a particular change or to change in general, the first-line manager would provide information repeatedly and sometimes focus more on the benefits for the staff over the benefits for the patients. The first-line managers suggested that they would give room to negative voices regarding a change or a new routine as a part of their strategy. Resistance had to be aired in order to be addressed, and they hoped that co-workers who were negative towards knowledge implementation would eventually accept the change.

The first-line managers also addressed **task-oriented leadership** components of implementation, such as procuring resources and providing education and information to their staff. The first-line managers’ priorities were described as proactive and deliberate, yet they had to both *act and react to support change*. Some actions necessarily were more of reactions, initiated by the context. For example, high staff turnover would create the need to focus on introducing new staff members to basic care procedures rather than on purposeful actions to implement new routines. The managers expressed it as starting over repeatedly and never getting a chance to evolve.

The managers described the changes associated with the expected CPG implementation in positive terms to their staff, hoping that the staff would then be more positive towards implementing the new or altered guideline. Yet they also described having to be certain and consistent regarding proposed changes, making it clear to the staff that these were not negotiable. Both their positive attitudes towards and firmness regarding the changes signified **change-oriented leadership**. The first-line managers made efforts to reach inter-professional consensus regarding new routines, but due to context restraints such as organizational structures or lack of direct authority over some professionals working outside the unit, this often meant they had to reach out to other managers in the organization in order to reach all professions. Co-workers that showed a willingness and ability to work with change processes were encouraged, even if their good intentions often fizzled out.

The managers stressed the importance of involving all staff when implementing a change, which was sometimes onerous. The first-line managers could find it challenging to manage implementation, particularly when they were short-staffed, and everyday patient care is always a priority. Even though they knew how to effectively facilitate a guideline implementation, they lacked sufficient time and staff. The first-line managers were concerned that directives to implement a CPG were sometimes issued without giving them adequate time to prepare. They also described high staff turnover as one of the context factors that presented the greatest challenge to implementation leadership, as competencies were lost when co-workers left. In their view, high staff turnover hampered their opportunities to implement improvements in care.

### The comprehensive understanding

First-line managers’ role in guideline implementation is signified by *balancing contexts*. Their work situation incorporates both an *outer and an inner context*, and they act in response to both contexts, adapting to and/or making adaptations, by *navigating the organization and its terms and conditions*. This represented a workplace that lacked an effective command or organizational structure, with unclear directives, lack of communication, and one-way communication from the top down. The first-line managers viewed navigating the organization and its terms and conditions as part of their role, albeit a frustrating part. The organizational structure was often unfit for nursing, limiting opportunities for multi-professional communication and cooperation. The managers felt that the organization was not prioritizing nursing issues and that they therefore had less power to influence health care because they were stuck in their units and were having a hard time making their voices heard.

The first-line managers’ outer context was represented by higher-level decision-makers, both within and outside of the organization. These were somewhat inflexible and commanding, and less susceptible to dialogue; they simply distributed guidelines and directives to be implemented. In this sphere, the first-line managers had little impact and found it hard to voice any concerns about what they were expected to accomplish. Yet the first-line managers *trusted that the directed guidelines were correct*, which helped them justify the directives when addressing their staff. In the inner context, the managers experienced more of a relational setting, with a stronger mandate and greater influence. Here, the first-line managers *acted and reacted to support change* by using different leadership behaviours based on local factors and on their experiences of what had worked in the past.

The first-line managers envisioned implementing new guidelines with a view to improving patient care, although this was not transparent in the directives they got. However, trusting that the upper-level management or external authorities had the same purpose with the guideline, the first-line managers invoked this vision to promote adoption and change. Within the inner context, the first-line managers took command of the implementation agenda and used their leadership role to interact with the staff so that communication about CPGs or proposed changes was no longer one-way.

**Fig. 1:**
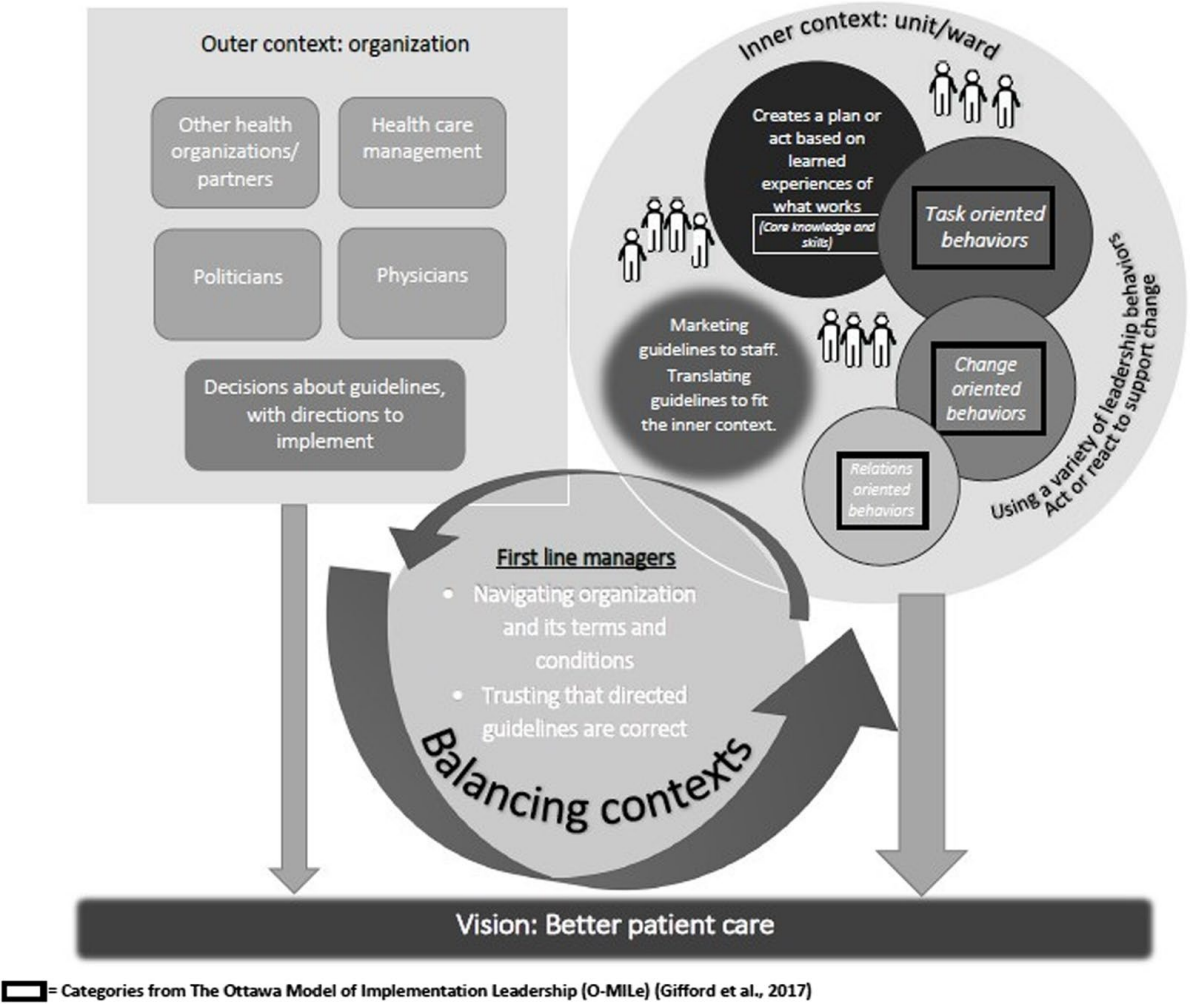
Visualisation of first-line managers experience of implementing guidelines

The comprehensive understanding is illustrated in Fig. 1, with correspondence to the structured analysis (OMILe and additional elements).

## Discussion

The first-line managers’ experience of CPG implementation in an orthopaedic care context illustrates a balancing act between contexts. Nursing and rehabilitation first-line managers in orthopaedic care navigate organization and its terms and conditions and describe a variety of leadership behaviours to facilitate guideline adoption and adherence. Yet contextual factors compel them to react rather than to employ purposeful actions. The first-line managers portrayed this as a conflict between what they considered optimal routes for ideal nursing and rehabilitation care of orthopaedic patients and what is conceivable in a busy everyday management role and context. The challenges of first-line management in EBP and guideline implementation will be addressed, along with what leadership support and development is needed for implementation leadership to sustain evidence-based nursing and rehabilitation.

First-line managers are positioned between upper management and clinical practice [9, 10]. As such, they have the potential to facilitate EBP. Through communication and by finding ways to bridge organizational barriers in order to motivate staff to see the patient outcomes of the new or altered guidelines, managers can create a shared vision of change. This requires navigating context and organizational conditions to explore the optimal routes to implementing new guidelines. Organizations often rely on the first-line managers’ ability to translate communication and visions mandated from the outer organization for staff’s acceptance of change [9]. This places the first-line managers in a central role: the progress of organizations depends heavily on their ability to make an ill-fitting guideline fit and to succeed with its implementation. Yet previous research suggests that lack of success in implementing EBPs could be explained by factors other than leadership [31, 32], which should take some of the pressure off the first-line managers’ shoulders. At the same time, there is support for the central role of the first-line managers regarding implementation in different settings [2, 8], leading to a perception of the importance of improving conditions for first-line managers to enable CPG implementation. In EBP implementation, leadership behaviours should be seen in the light of both the culture and the organization where the leadership is enacted [33], while at the same time leadership itself is often considered to be a part of the context [34].

The first-line managers in our study described numerous behaviours to support change originating from their previous experiences of successful practice. They also described the barriers they had identified when working with change processes. The behaviours illustrated have been suggested to be effective components in implementation leadership [27], but there is still a need for a better understanding of the timing of such behaviours as well as of how context interplays with them. In contrast to what has hitherto been suggested as managers’ proactive and targeted conduct, our findings indicate that first-line managers’ reactions in favour of guideline implementation is unconscious and intuitive. Rather than planning and defining their actions by means of an explicit understanding of implementation processes, the managers described acting in accordance with what they deemed most favourable for implementation, based on know-how and experience, as well as what they considered appropriate and possible given the context. First-line managers’ struggle to operate as facilitators for EBP implementation in an incompatible context with opposing demands [31]. Consequently, we suggest that training and supporting first-line managers in knowledge implementation recognizes their prior experiences, reinforcing what has served well, in what context, and for whom, yet re-establishing leadership strategies that might be more effective for promoting evidence-based practice.

Even with such skills, the managers described barriers that prevented them from acting in ways they thought would be right. Rather, actions more often became *reactions* because of the organization and its terms and conditions. Adapting to staff shortages or to high staff turnover, for example, led the first-line managers to put more efforts into certain leadership behaviours, such as procuring resources, rather than into what they actually considered more important in order to reach implementation success. This indicates that first-line managers might have unused competencies regarding EBP implementation and thus are underused as resources. Context factors have been identified as having a great impact when it comes to implementation in health care [34–36], and recent research highlights how first-line managers have applied situational work in order to handle the complexity of implementing changes in the public sector [37]. Knowing the importance of developing EBP while lacking sufficient resources to do so can be stressful for the first-line managers [38]. However, even though first-line managers understand the importance of their behaviours when it comes to facilitating the implementation of CPGs, they cannot always use them [39]. This indicates great potential for further support and training for first-line managers as well as a continued need for organizational support and leadership educational programmes with a focus on implementation leadership skills. With better conditions for first-line managers to enact implementation leadership, further opportunities for more evidence-based care can advance.

### Methodological considerations/Study limitations

This study represents first-line managers engaged in the OPTION trial, and thus a large share of the orthopaedic care first-line managers across Sweden. Yet it still represents only a minor part of all nursing and rehabilitation managers across Sweden and globally. While the experiences shared are similar to those described in previous studies (such as [31] and [32]), this study has identified novel perspectives contributing to the growing understanding of first-line managers’ situation, conditions, and needs. These contemporary findings could be used to inform the development of validated leadership surveys in order to reach a broader population of first-line nursing and/or rehabilitation managers. To date, there are few such instruments for measuring implementation leadership available in Swedish and/or for the Swedish healthcare context. The Implementation Leadership Scale (ILS) [40] incorporates many, if not all, aspects identified in this study, but further development would be helpful.

## Conclusion

This study illustrates first-line managers’ experience of implementing CPGs in orthopaedic nursing and rehabilitation care – a process in which they balance the outer and inner contexts. Their behaviours are intended to move forward with CPG implementation, and their position provides opportunities to enhance EBP, even if their full use of leadership is impaired by the organization and its limited resources.

The first-line managers described leadership behaviours that had previously proved effective for implementation leadership, here employed as a result of their navigation of their contexts and along with the everyday conditions of acute care. While the first-line managers meant to proceed with the CPG implementation, they balanced the often sub-optimal conditions for the benefit of adoption of and adherence to clinical practice guidelines. Further opportunities to plan and perform deliberate behaviours are needed, with strategies aiding first-line managers to facilitate implementation.

## Data Availability

The data that support the findings of this study are available on request from the corresponding author (EF), upon reasonable request. The data are not publicly available as it contains information that could compromise the privacy of research participants.

## Abbreviations

CPG: Clinical Practice Guideline
EBP: Evidence Based Practice
O-MILe: The Ottawa Model of Implementation Leadership
OPTION: Onset PrevenTIon of urinary retention in Orthopaedic Nursing and rehabilitation
